# Assessment of Metal Concentrations and Associations with Pulmonary Function among Children with Asthma in Chicago, Illinois

**DOI:** 10.3390/ijerph18147279

**Published:** 2021-07-07

**Authors:** Jessica M. Madrigal, Victoria Persky, Brian P. Jackson, Amy Bain, Matt Siemer, Andrea A. Pappalardo, Maria Argos

**Affiliations:** 1 Division of Epidemiology and Biostatistics, School of Public Health, University of Illinois Chicago, Chicago, IL 60612, USA; jmadri1@uic.edu (J.M.M.); vwpersky@uic.edu (V.P.); 2 Department of Earth Sciences, Dartmouth College, Hanover, NH 03755, USA; Brian.P.Jackson@Dartmouth.edu; 3 Mobile Care Chicago, Chicago, IL 60609, USA; amyjbain@outlook.com (A.B.); msiemer@mobilecarefoundation.org (M.S.); 4 Department of Pediatrics, College of Medicine, University of Illinois Chicago, Chicago, IL 60612, USA; apappa2@uic.edu; 5 Department of Medicine, College of Medicine, University of Illinois Chicago, Chicago, IL 60612, USA

**Keywords:** metals, pulmonary function, respiratory function, asthma, children

## Abstract

Individuals living in areas with the potential for elevated metal exposure from industrial sources may have reduced pulmonary function. We evaluated cross-sectional associations of toenail concentrations of 17 metals within a community area of residence and asthma control in 75 children, and pulmonary function measures [forced expiratory volume in one second (FEV1; liters), forced vital capacity (FVC; liters), FEV1 to FVC ratio (FEV1:FVC), and mid-exhalation forced expiratory flow rate (FEF 25–75%; liters/second)], in a subsample of 39 children with diagnosed asthma in Chicago, Illinois. Linear regression models were used to estimate adjusted regression coefficients and standard errors (SE) for the associations between ≥ median versus <median metal exposures and natural log-transformed (ln) pulmonary function test parameters. Toenail levels of cadmium, cobalt, iron, manganese, and vanadium were higher among children residing near an industrial corridor than those in a comparison community. Copper concentrations were inversely associated with lnFEV1 (β = −0.10, SE = 0.04, *p* = 0.01), lnFEV1:FVC (β = −0.07, SE = 0.03, *p* = 0.02) and lnFEF 25–75% (β = −0.25, SE = 0.09, *p* = 0.01); manganese concentrations were inversely associated with lnFEV1 (β = −0.11, SE = 0.04, *p* = 0.01), lnFEV1:FVC (β = −0.07, SE = 0.03, *p* = 0.02), and lnFEF 25–75% (β = −0.28, SE = 0.10, *p* = 0.004), and vanadium concentrations were inversely associated with lnFEV1 (β = −0.08, SE = 0.04, *p* = 0.05) and lnFVC (β = −0.07, SE = 0.03, *p* = 0.03). Nickel and copper were associated with uncontrolled asthma (OR = 6.8; 95% CI 2.0, 22.8 and OR = 4.6; 95% CI 1.0, 21.0, respectively). These data suggest that selected metal exposures may be associated with impaired pulmonary function parameters and reduced asthma control among children with preexisting asthma.

## 1. Introduction

Asthma affects approximately six million children in the United States [1]. It is more prevalent among boys, among children who are African American or Puerto Rican, and among children from low-income households [1]. Even with recent developments in childhood asthma therapy, the overall age-adjusted rate of asthma emergency department (ED) visits among children aged 18 years or younger in Chicago was 126.9 per 10,000 population in 2017 [2], with noticeable variation by geographic location. In communities near the Calumet Industrial Corridor in Chicago, the age-adjusted rate of ED visits for asthma varies from 170.1 to 179.2 per 10,000 population for children aged under 18 years. This contrasts with other Chicago community areas that are demographically similar but not situated adjacent to the industrial corridor, where the rates range from 52.8 to 96.9 per 10,000 population [2].

Although asthma is a chronic lung disease, reducing exposures to environmental irritants and allergens can decrease asthma symptoms. Previous studies have linked metal exposures to childhood asthma, the severity of symptoms, and decreased pulmonary function [3,4,5,6,7,8,9]. This linkage suggests that reduced exposure to metals may be effective in reducing morbidity from the disease. Investigations of the effects of metals on pulmonary function in children have examined children in general populations [10,11,12,13], as well as children living near e-waste facilities [3,14], in areas of high arsenic [15] or lead exposures [4,7,16,17], and in an area of ferroalloy industries [5]. However, results from these studies may have varied, in part, due to different patterns of co-exposure or mixtures of metal exposures.

The city of Chicago offers a unique opportunity to examine associations of metals with respiratory function due to known geographic variability in metal exposures from traffic and industry. The present study focused on children residing near the Calumet Industrial Corridor and a demographically similar community area without the same industrial exposures. The Calumet Industrial Corridor occupies approximately 4000 acres in southeast Chicago, adjacent to residential community sites. This area has a long history of steel production, and the current industry includes metal processing and recycling plants, storage of petroleum coke, arc furnace dust, and other metal products. The objectives of the present study were to characterize levels of toxic and trace metals in toenail clippings obtained from children with asthma attending grade schools near the industrial corridor compared to children with asthma in a demographically similar community area in Chicago and to examine associations of toenail metal concentrations with pulmonary function parameters and asthma control.

## 2. Materials and Methods

### 2.1. Study Population

In partnership with Mobile Care Chicago—a community-based, non-profit organization that provides asthma care for children in under-served and under-resourced community areas throughout Chicago—we identified two community areas for the recruitment of study participants. One community area was adjacent to the industrial corridor, and the other community area was demographically comparable but located elsewhere in Chicago. Eligibility criteria for participation in the study included children receiving asthma care from Mobile Care Chicago and enrolled in grades 3 to 8 of an elementary school in one of the two selected community areas. Children and their parents/guardians were invited to participate in the study during a regularly scheduled asthma care visit. Written informed consent and assent were obtained from the parent/guardian and youth, respectively. The study was approved by the University of Illinois Chicago Institutional Review Board. Between November 2016 and April 2018, 78 schoolchildren (42 from the community area adjacent to the industrial corridor and 36 from the comparison community area) were enrolled in the study. For these analyses, we excluded an additional three participants with six or more metal concentrations below the limit of detection (two from the adjacent and one from the comparison community area), presumed to be the result of insufficient toenail clipping sample mass. Of those, 39 participants were judged as having an acceptable spirometry reading for lung function analyses according to the American Thoracic Society (ATS) criteria [18] as determined by an expert clinical reviewer (A.P.), of which 22 were from the community area adjacent to the industrial corridor and 17 from the comparison community area.

### 2.2. Outcome Assessment

Participants completed spirometry testing as part of their clinical asthma visit with Mobile Care Chicago. Spirometry was performed using the Orbit portable spirometer (QRS Diagnostic, Maple Grove, MN, USA). Spirometry was conducted in the standing position following a standardized protocol according to the recommendations of the American Thoracic Society (ATS) [18]. Participants aged 6 to 10 years were asked to exhale for a minimum of 3 s, while those aged 11 years and older were asked to exhale for 6 s. Participants repeated the test until they could achieve an acceptable and reproducible spirogram for a maximum of three attempts. The three spirometry readings were recorded for each participant. We abstracted pulmonary function data from the best spirogram for forced expiratory volume in one second (FEV1; liters), forced vital capacity (FVC; liters), FEV1 to FVC ratio (FEV1:FVC), and mid-exhalation forced expiratory flow rate (FEF 25–75%; liters/second), selected by clinical expert review (A.P.) independent of knowledge of toenail metal concentrations. Percent predicted values for FVC, FEV1, and FEF 25–75% were calculated based on participant age, sex, and race/ethnicity using the criteria outlined by Hankinson and colleagues [19]. Asthma control was determined by the Asthma Control Test (ACT) [20].

### 2.3. Exposure Assessment

Participants provided toenail clipping samples for the measurement of metal concentrations. Participants were instructed to (1) clip toenails from all toes, (2) clip the amount with which they were comfortable, and (3) place and seal all toenail clippings in the provided envelope pre-labeled with their unique study identifier. Toenail clippings were stored at room temperature until shipment for analysis. Quantification of toenail metal concentrations was performed at the Dartmouth College Trace Element Analysis Laboratory. Toenail samples were washed at the laboratory to remove any visible dirt or nail polish using a standard protocol. Cleaned nail samples were weighed before microwave digestion, and toenail metal concentrations were measured using inductively coupled plasma-mass spectrometry (ICP-MS). Concentrations of aluminum (Al), chromium (Cr), manganese (Mn), iron (Fe), cobalt (Co), nickel (Ni), copper (Cu), zinc (Zn), arsenic (As), selenium (Se), molybdenum (Mo), cadmium (Cd), tin (Sn), antimony (Sb), mercury (Hg), vanadium (V), and lead (Pb) were measured in micrograms per gram (µg/g) from all samples. Any concentration below the instrument limit of detection was flagged, and the instrument inferred the concentration from the experiment’s calibration curve. For the 75 samples, values for cadmium (*n* = 4, 5.3%), chromium (*n* = 1, 1.3%), mercury (*n* = 29, 38.7%), molybdenum (*n* = 11, 14.7%), antimony (*n* = 1, 1.3%), and vanadium (*n* = 30, 40.0%) were imputed. Toenail metal concentrations were dichotomized at the median based on each metal concentration distribution in the present analyses for the overall group of 75 for comparisons between community areas and asthma control status and for the group of 39 children for analyses of pulmonary function, with the low exposure category serving as the reference group.

### 2.4. Covariates

Parent/guardian-reported demographic information for the child was collected, including age (years), race/ethnicity (non-Hispanic White, non-Hispanic Black, Mexican American, other Hispanic, or other/unknown), sex (male/female), name of school attending, and grade level. On the day of the study visit, the Mobile Care Chicago provider recorded height (inches) and weight (pounds). Body mass index (BMI, kg/m^2^) was subsequently calculated.

### 2.5. Statistical Analysis

Selected participant characteristics were summarized using frequencies and percentages. Continuous variables were described using mean ± standard deviation (SD) or median (interquartile range, IQR) for skewed variables. Metal distributions were compared using the two-sample Wilcoxon rank sum test. Spearman’s rank correlation coefficients evaluated correlations among the toenail metal concentrations.

We evaluated associations of each metal concentration with measures of lung function, including FEV1 (liters), FVC (liters), FEV1:FVC ratio, and FEF 25–75% (liters/second) and the corresponding percent predicted pulmonary function parameters. All pulmonary function measures were modeled as continuous outcome variables using linear regression models. Skewed outcome distributions observed for pulmonary function measures in liters and liters/second were natural log-transformed before modeling. Age (continuous, years), sex (male, female), race/ethnicity (non-Hispanic, Hispanic), BMI (continuous, kg/m^2^), height (continuous, centimeters), school attended, and community area (adjacent to the industrial corridor, comparison) were included in all models of pulmonary function tests in liters and liters/second. Models for predicted pulmonary function parameters were adjusted for BMI (continuous, kg/m^2^), height (continuous, centimeters), school attended, and community area (adjacent to the industrial corridor, comparison). As a sensitivity analysis, we compared model results using the full sample to model results restricted to participants who identified as Hispanic (*n* = 35). An additional sensitivity analysis was performed using generalized estimating equations (GEE) to account for clustering by school using an exchangeable correlation matrix, adjusted for the covariates described above.

Uncontrolled asthma (ACT score ≤ 19) [21] was modeled using GEE, with a binomial distribution and a logit link function, to account for clustering by the school using an exchangeable correlation matrix, adjusted for age (continuous years), sex (male/female), BMI (continuous, kg/m^2^), and community area (adjacent to the industrial corridor, comparison). Due to limitations in model convergence, we could not adjust for race/ethnicity in the models for uncontrolled asthma. As such, we compared model results using the full sample to model results restricted to participants who identified as Hispanic (*n* = 70). All analyses were conducted in SAS version 9.4 (Cary, NC, USA).

## 3. Results

Among the 75 participants with available toenail metal concentrations, the average age was 10 years (±1 year SD), and 63% were male. The majority of participants (93%) identified as Hispanic, and 87% reported a Mexican background. Approximately half (53%) of the study sample was enrolled from the community area adjacent to the industrial corridor. Uncontrolled asthma based on ACT score ≤19 was observed in approximately 21% of the overall sample. Among the subsample with available spirometry, the median FEV1, FVC, FEV1:FVC, and FEF 25–75% values for study participants were 2.0 L (IQR 0.7), 2.3 L (IQR 0.9), 0.88 (IQR 0.1), and 2.3 L per second (IQR 1.1). Details by community area are presented in Table 1.

The distributions of toenail metal concentrations overall and by community area are shown in Table 2. Toenail concentrations of cadmium, cobalt, iron, manganese, and vanadium were significantly higher in children residing near the industrial corridor, while mercury and zinc were significantly lower. Correlations between the metals are shown in Supplementary Materials Table S1. Moderate correlations >0.60 were observed among cobalt and nickel (r_S_ = 0.63), cobalt and chromium (r_S_ = 0.64), cobalt and iron (r_S_ = 0.70), and iron and nickel (r_S_ = 0.81).

### 3.1. Pulmonary Function Parameters

Associations between toenail metal concentrations and natural log-transformed pulmonary function measures are summarized in Table 3. In adjusted models, toenail copper concentration was inversely associated with FEV1 (*p* = 0.01), FEV1:FVC (*p* = 0.02), and FEF 25–75% (*p* = 0.01). Toenail manganese concentration was inversely associated with FEV1 (*p* = 0.01), FEV1:FVC (*p* = 0.02), and FEF 25–75% (*p* = 0.004). Toenail cadmium concentration was inversely associated with FEV1 (*p* = 0.08). Toenail cobalt concentration was inversely associated with FEV1:FVC (*p* = 0.04). Toenail vanadium concentration was inversely associated with FEV1 (*p* = 0.05) and FVC (*p* = 0.03), and toenail molybdenum was inversely associated with FVC (*p* = 0.09). Toenail selenium concentration was inversely associated with FVC (*p* = 0.01) and positively associated with FEV1:FVC (*p* = 0.09). Toenail nickel concentration was positively associated with FVC (*p* = 0.04). In sensitivity analyses restricted to the 35 children with self-reported Hispanic ethnicity, associations were similar, except that selenium was no longer associated with FEV1:FVC (Supplementary Materials Table S2).

In analyses modeling percent predicted pulmonary function parameters, toenail copper concentration was inversely associated with FEV1% (*p* = 0.02), FEV1:FVC% (*p* = 0.05), and FEF 25–75% (*p* = 0.01) (Supplementary Materials Table S3). Toenail manganese concentration was inversely associated with FEV1% (*p* = 0.02), FEV1:FVC% (*p* = 0.06), and FEF 25–75% (*p* = 0.004); toenail molybdenum concentration was inversely associated with FVC% (*p* = 0.06); and toenail vanadium concentration was inversely associated with FEV1% (*p* = 0.02) and FVC% (*p* = 0.01). Cadmium and cobalt were no longer associated with pulmonary function measures, while toenail selenium concentration was inversely associated with FVC% (*p* = 0.01) and positively associated with FEV1:FVC% (*p* = 0.08). Toenail nickel concentration was positively associated with FVC% (*p* = 0.05) and toenail lead concentration was positively associated with FEV1:FVC% (*p* = 0.07). In sensitivity analyses restricted to the 35 children with self-reported Hispanic ethnicity, associations were similar (Supplementary Materials Table S4). Overall findings were also similar in sensitivity analyses using GEE (data not shown).

### 3.2. Asthma Control

As shown in Table 4, toenail nickel (OR 6.8; 95% CI: 2.0, 22.8) and copper (OR 4.6; 95% CI: 1.0, 21.0) concentrations were associated with uncontrolled asthma. In sensitivity analyses restricted to the 70 children with self-reported Hispanic ethnicity, associations did not substantively change (Supplementary Materials Table S5).

## 4. Discussion

This study characterized toxic and trace metals in toenail samples obtained from 75 children and youth with asthma residing in Chicago. Toenail concentrations of cadmium, cobalt, iron, manganese, and vanadium were significantly higher, and mercury and zinc significantly lower in children living near the industrial corridor. However, median toenail concentrations of cadmium, cobalt, vanadium, and mercury were less than 0.05 µg/g in the study sample overall. We observed positive associations of toenail copper and nickel concentrations with the prevalence of uncontrolled asthma. Among the 39 children with available pulmonary function test data, we observed inverse associations of toenail, copper, vanadium, and manganese with select pulmonary function parameters in analyses using liters and percent predicted measures.

Although environmental exposures are related to childhood asthma, few studies have examined pulmonary function or asthma control with specific metal exposures. Those that have studied pulmonary function or respiratory symptoms with measured metal exposures in children have not been consistent. In a previous cross-sectional study of blood and urinary concentrations of cadmium, cobalt, lead, and manganese in a sample of 1234 children aged 6–17 years from the 2011–2012 NHANES, blood manganese concentration was inversely associated with FEV1 and FVC among older youth [10]. In that same study, urinary manganese concentration was inversely associated with FEV1:FVC and FEF 25–75%, and urinary lead concentration was inversely associated with FEF 25–75% [10]. In a study of Korean children, blood mercury concentration measured at ages 7 to 8 was associated with an increased risk of asthma and wheezing at ages 11 to 12 [9]. In contrast, a study of 358 healthy children in Mexico found that urinary arsenic concentration was inversely associated with FVC. Children with a restrictive lung pattern had higher urinary arsenic levels than those with normal lung function patterns [15]. In contrast, nail selenium and zinc were lower in adolescents with asthma in Brazil [8]. In a study of 65 children with recurrent wheezing, hair cadmium concentration >0.17 µg/kg was associated with higher odds of having three or more wheezing episodes in the prior six months relative to those with hair cadmium concentration ≤0.17 µg/kg [22]. In a Turkish study of 42 children with asthma and 30 healthy controls, plasma levels of iron were higher, but plasma levels of selenium and manganese were lower, and copper levels did not differ among children with asthma compared to controls [23].

The previous literature suggests possible associations of lead with pulmonary function. In the present study, toenail lead concentration was not associated with pulmonary function parameters in analyses using liters and was positively associated with percent predicted measures of FEV1:FVC (*p* = 0.07) but not with other pulmonary function measurements. In a study of 373 Polish schoolchildren, blood lead levels were inversely associated with FVC [13]. Blood lead levels were similarly associated with asthma in 930 kindergarten students in Taiwan [24] and 356 children in Michigan [6], but not among children in Missouri [17]. Among 1,788 children in the 2005–2006 NHANES, blood lead was not associated with asthma [16]; however, analyses of the 2007–2012 NHANES survey cycles showed that children with high blood lead had a higher prevalence of asthma [4].

Longitudinal studies have suggested that metals may increase the incidence of respiratory symptoms and decrease pulmonary function. Higher levels of ambient nickel and vanadium measured in PM_2.5_ were associated with an increased probability of wheeze among young children (<2 years old) in New York City [11]. A panel study of 43 school children in grades 3–6 in Korea found a reduction in peak expiratory flow rate with higher exposure to manganese and lead [25]. In comparison, a panel study of 70 asthmatic school children in Canada, in whom 217 measures of personal exposure were obtained, found that exposures to iron and aluminum contained in fine particulate matter (PM_2.5_), but not to other metals, were associated with higher levels of fractional expired nitric oxide, a marker related to pulmonary inflammation [26].

The few studies of children near industrial exposure sources have found higher metal exposures related to respiratory outcomes. Studies of Chinese preschool children near an e-waste facility showed positive associations of blood lead and cadmium levels with decreased FVC and FEV1, as well as associations of nickel and manganese with decreased FVC [3]. Among 410 adolescents living near an industrial area in Italy, concentrations of manganese, nickel, and chromium from PM_10_ were significantly and positively associated with parent-reported asthma [5]. In that same study, concentrations of manganese and nickel were associated with asthma medications’ reported use within the prior 12 months [5].

It is difficult to compare our findings with previous studies given the variations in mixtures of exposures, biological samples assessed, and populations studied. Among studies in children that have examined metals with pulmonary function [3,10,12,13,14,17], the three papers that included manganese found inverse associations of manganese with measures of respiratory function [10,12,14]. One study that included urinary cobalt found no association with pulmonary function [10]. In the present study, toenail cobalt concentration was inversely associated with FEV1: FVC. Among studies reporting relationships of metal exposure with asthma symptoms or control [7,11,22,26], one showed a positive association of exposure to ambient concentrations of nickel measured in PM_2.5_ and wheezing, whereas one showed no association of PM_2.5_ concentrations of nickel with expired nitric oxide. In our analyses of pulmonary function measures, we observed a positive association of toenail nickel concentration with FVC; although, we also found that toenail nickel concentration was associated with higher, rather than lower, odds of uncontrolled asthma, which is consistent with prior reports of associations of nickel sensitivity and allergy with the disease [27,28,29]. To the best of our knowledge, we are the first to report associations with toenail copper concentrations. No prior published studies have related copper with asthma control. Inconsistencies in study findings may be related to exposure assessment, variations in exposure levels, and differences in study endpoints, confounders, or effect modifiers.

In our study of children with asthma, we observed inverse associations for measures of copper, manganese, and vanadium both in analyses using liters/second and those using percent predicted pulmonary function. Inverse associations of selenium and FVC, and positive associations of selenium with FEV1:FVC were consistent in analyses using liters/second and those using percent predicted pulmonary function as well, although in both analyses, the positive association with FEV1:FVC was of only borderline significance. Some of the inverse associations we observed were with FEF 25–75, which may be an indicator of patency and inflammation in the small airways [30,31,32,33] and a sensitive marker of symptomatic disease in children with asthma [33,34,35]; however, the use of this marker is still controversial given its lack of reproducibility. Therefore, interpretation of these findings awaits further studies. We also observed inverse associations with the FEV1:FVC ratio, which measures the degree of airflow obstruction and has been associated with symptom severity [36,37]. If the inverse associations we observed in this study are confirmed, this may indicate that exposure to metals, such as copper, manganese, and vanadium may contribute to decreased pulmonary function among children with preexisting asthma.

We also observed toenail zinc levels that were significantly lower in children living near the industrial corridor, which may suggest a zinc deficiency in these children compared to the comparison group. The mechanisms by which metals may exacerbate respiratory disease are not well established. Metal exposures are known to be associated with inflammatory markers and measures of oxidative stress [38,39,40], which, in turn, have been implicated in the pathogenesis of asthma [41,42,43]. Future studies are needed to delineate sources of exposure to metals in the environment among children living in urban areas and their contribution to asthma, as well as the biologic pathways by which metal exposures are operative.

Our study’s strengths include the use of objective measures of both toenail metal concentrations and pulmonary function tests based on spirometry performed in a clinical setting. This study’s limitations include the relatively small number of participants, which did not permit us to employ analytical approaches to examine metal mixtures. Our model-based estimates may have limited power due to the small sample size we have available. We acknowledge that a larger sample size would offer more insights into the associations under study. We did not account for multiple comparisons in this study since we evaluated pre-specified hypotheses. Additionally, data on participant medications for asthma control were not available. Exposure to other unmeasured pollutants correlated with the metals measured in this study may have confounded our analyses; however, community area was included as a covariate in all analyses, which accounted to some extent for community-level confounders. Toenail clippings were washed before analysis to minimize potential contamination from the stainless steel clippers used to collect the samples, but some exposure misclassification may still be present. A longitudinal assessment of pulmonary function would additionally strengthen the findings of this study. Nevertheless, this is one of the few studies examining the effects of residential exposures near industrial sites with pulmonary function among children with asthma. Confirmation of the findings in future studies would be necessary for defining intervention strategies.

## 5. Conclusions

Our findings suggest that children with asthma living in an urban area have measurable toenail concentrations of toxic and trace metals, as evidenced by the levels measured in toenail biospecimens in our study. We show that children residing near an industrial corridor have higher toenail concentrations of cadmium, cobalt, iron, manganese, and vanadium compared to children with asthma in a demographically similar community area in Chicago. This study indicates that toenail concentrations of vanadium, copper, and manganese may be inversely associated with pulmonary function test parameters, and copper and nickel may be positively associated with uncontrolled asthma. Future longitudinal studies are needed to confirm these findings and expand our understanding of how environmental metal exposures may impact children’s environmental health.

## Figures and Tables

**Table 1:** Selected characteristics of study participants by community area, comparing the full sample (*n* = 75) to those with available spirometry (*n* = 39).

Characteristic	Comparison Community Area		Industrial Corridor Adjacent Community Area	
	Full Sample *n* = 35	Spirometry Subsample *n* = 17	Full Sample *n* = 40	Spirometry Subsample *n* = 22
**Age (years)**	*n* (%)	*n* (%)	*n* (%)	*n* (%)
7–10	16 (45.7)	9 (52.9)	21 (52.5)	16 (72.7)
11–15	19 (54.3)	8 (47.1)	19 (47.5)	6 (27.3)
**Sex**				
Male	19 (54.3)	9 (52.9)	28 (70.0)	16 (72.7)
Female	16 (45.7)	8 (47.1)	12 (30.0)	6 (27.3)
**Race/ethnicity**				
Hispanic	35 (100.0)	17 (100.0)	35 (87.5)	18 (81.8)
non-Hispanic	0	0	5 (12.5)	4 (18.2)
**ACT score**, Median (IQR)	23 (4)	23 (6)	23 (4)	24 (5)
≤19 (uncontrolled)	7 (20.0)	3 (17.7)	9 (22.5)	4 (18.2)
>19 (controlled)	28 (80.0)	14 (82.3)	31 (77.5)	18 (81.8)
**Pulmonary function parameters**				
*% predicted*		**Mean ± SD**		**Mean ± SD**
FEV1		93 ±14		92 ±11
FVC		94 ±12		92 ±9
FEV1:FVC		98 ±10		99 ±8
FEF 25–75%		93 ±30		92 ±26
*Liters*		**Median (IQR)**		**Median (IQR)**
FEV1		2.1 (0.5)		1.9 (0.6)
FVC		2.6 (0.9)		2.1 (0.7)
FEV1:FVC		0.9 (0.1)		0.9 (0.1)
FEF 25–75%		2.7 (1.0)		2.2 (1.1)

Forced expiratory volume in one second (FEV1), forced vital capacity (FVC), FEV1 to FVC ratio (FEV1:FVC), and mid-exhalation forced expiratory flow rate (FEF 25–75%).

**Table 2:** Distribution of toenail metal concentrations, overall and by community area (*n* = 75).

Characteristic	Overall (*n* = 75)	Comparison Community Area (*n* = 35)	Adjacent Community Area (*n* = 40)	*p* Value ^a^
Exposure Levels, µg/g	Median (Interquartile Range)			
Aluminum	12.3 (7.3)	12.3 (13.1)	12.4 (6.9)	0.64
Arsenic	0.15 (0.28)	0.13 (0.23)	0.16 (0.31)	0.27
Cadmium	0.03 (0.04)	0.02 (0.02)	0.04 (0.06)	<0.01
Cobalt	0.04 (0.05)	0.03 (0.04)	0.04 (0.08)	0.04
Chromium	0.29 (0.36)	0.29 (0.35)	0.28 (0.44)	0.71
Copper	4.5 (1.6)	4.7 (1.8)	4.4 (1.6)	0.19
Iron	51.2 (85.8)	32.5 (53.3)	80.0 (138.0)	0.01
Mercury	0.03 (0.03)	0.03 (0.05)	0.02 (0.02)	0.03
Manganese	1.1 (1.4)	0.57 (0.99)	1.4 (1.4)	<0.01
Molybdenum	0.02 (0.02)	0.02 (0.02)	0.02 (0.03)	0.78
Nickel	13.4 (35.4)	8.4 (33.9)	16.2 (52.0)	0.18
Lead	1.2 (1.7)	1.4 (2.4)	1.0 (1.0)	0.24
Antimony	0.14 (0.14)	0.11 (0.25)	0.15 (0.10)	0.15
Selenium	0.87 (0.17)	0.88 (0.26)	0.84 (0.17)	0.25
Tin	0.27 (0.23)	0.35 (0.18)	0.25 (0.22)	0.07
Vanadium	0.02 (0.04)	0.01 (0.03)	0.02 (0.10)	<0.01
Zinc	96.8 (27.2)	106.2 (25.8)	90.5 (20.9)	<0.01

^a^ Two-sided *p*-value comparing the comparison and industrial corridor community areas using the Wilcoxon two-sample test.

**Table 3:** Associations between toenail metal concentrations and pulmonary function parameters among children with asthma (*n* = 39).

Exposure (µg/g)	ln (FEV1, L) ^a^	ln (FVC, L) ^a^	ln (FEV1:FVC) ^a^	ln (FEF 25–75, L/sec) ^a^
	β (SE)	β (SE)	β (SE)	β (SE)
Adjacent Community	0.01 (0.06)	0.01 (0.06)	−0.002 (0.04)	−0.03 (0.15)
*p* value	0.89	0.87	0.96	0.83
Aluminum ≥ 13.4	−0.03 (0.04)	0.02 (0.04)	−0.05 (0.03)	−0.13 (0.10)
*p* value	0.46	0.68	0.11	0.18
Arsenic ≥ 0.15	−0.004 (0.04)	0.04 (0.04)	−0.04 (0.03)	−0.13 (0.09)
*p* value	0.92	0.31	0.18	0.15
Cadmium ≥ 0.03	**−0.07 (0.04)**	−0.03 (0.04)	−0.04 (0.03)	−0.14 (0.10)
*p* value	**0.08**	0.37	0.17	0.13
Cobalt ≥ 0.05	−0.02 (0.04)	0.04 (0.04)	**−0.06 (0.03)**	−0.16 (0.10)
*p* value	0.65	0.29	**0.04**	0.12
Chromium ≥ 0.31	−0.03 (0.04)	0.005 (0.04)	−0.03 (0.03)	−0.06 (0.09)
*p* value	0.53	0.89	0.28	0.53
Copper ≥ 4.5	**−0.10 (0.04)**	−0.03 (0.04)	**−0.07 (0.03)**	**−0.25 (0.09)**
*p* value	**0.01**	0.34	**0.02**	**0.01**
Iron ≥ 53.9	0.01 (0.05)	0.01 (0.04)	−0.004 (0.03)	−0.03 (0.10)
*p* value	0.89	0.80	0.91	0.74
Mercury ≥ 0.03	−0.05 (0.05)	−0.04 (0.04)	−0.01 (0.03)	−0.08 (0.11)
*p* value	0.28	0.34	0.67	0.48
Manganese ≥ 1.1	**−0.11 (0.04)**	−0.04 (0.04)	**−0.07 (0.03)**	**−0.28 (0.10)**
*p* value	**0.01**	0.34	**0.02**	**0.004**
Molybdenum ≥ 0.02	−0.02 (0.04)	**−0.06 (0.04)**	0.03 (0.03)	0.03 (0.10)
*p* value	0.56	**0.09**	0.27	0.75
Nickel ≥ 15.2	0.04 (0.04)	**0.07 (0.04)**	−0.03 (0.03)	−0.05 (0.10)
*p* value	0.38	**0.04**	0.25	0.64
Lead ≥ 1.2	−0.003 (0.05)	−0.05 (0.04)	0.04 (0.03)	0.08 (0.11)
*p* value	0.95	0.25	0.18	0.45
Antimony ≥ 0.14	0.003 (0.05)	0.01 (0.04)	−0.003 (0.03)	−0.04 (0.11)
*p* value	0.95	0.90	0.92	0.69
Selenium ≥ 0.88	−0.04 (0.05)	**−0.09 (0.04)**	**0.05 (0.03)**	0.08 (0.10)
*p* value	0.35	**0.01**	**0.09**	0.47
Tin ≥ 0.27	0.04 (0.04)	0.008 (0.04)	0.03 (0.03)	0.13 (0.10)
*p* value	0.38	0.83	0.31	0.18
Vanadium ≥ 0.02	**−0.08 (0.04)**	**−0.07 (0.03)**	−0.01 (0.03)	−0.10 (0.10)
*p* value	**0.05**	**0.03**	0.84	0.28
Zinc ≥ 98.8	−0.03 (0.04)	−0.02 (0.04)	−0.01 (0.03)	−0.05 (0.10)
*p* value	0.48	0.55	0.79	0.64

^a^ Adjusted for sex, age (continuous, years), race/ethnicity (Hispanic, non-Hispanic), body mass index (continuous, kg/m^2^), height (continuous, centimeters), community area (adjacent, comparison), and school. *p* value < 0.1 shown in bold text.

**Table 4:** Associations between toenail metal concentrations and asthma control among children with asthma (*n* = 75).

Exposure (µg/g)	Uncontrolled Asthma	Controlled Asthma	OR (95% CI) ^a^
	ACT ≤19	ACT >19	
	(*n* = 16)	(*n* = 59)	
	N (%)	N (%)	
Adjacent Community	9 (56.3)	31 (52.5)	1.1 (0.4, 3.6)
Aluminum ≥ 12.3	10 (62.5)	27 (45.8)	2.0 (0.6, 5.9)
Arsenic ≥ 0.15	10 (62.5)	27 (45.8)	1.8 (0.5,5.9)
Cadmium ≥ 0.03	9 (56.3)	28 (47.5)	1.4 (0.4, 4.5)
Cobalt ≥ 0.04	10 (62.5)	27 (45.8)	2.3 (0.6, 9.3)
Chromium ≥ 0.29	9 (56.3)	28 (47.5)	1.5 (0.2, 9.7)
Copper ≥ 4.5	12 (75.0)	26 (44.1)	**4.6 (1.0, 21.0)**
Iron ≥ 51.2	11 (68.8)	27 (45.8)	3.2 (0.8, 13.6)
Mercury ≥ 0.03	9 (56.3)	29 (49.2)	1.5 (0.4, 5.6)
Manganese ≥ 1.1	9 (56.3)	29 (49.2)	1.3 (0.3, 4.7)
Molybdenum ≥ 0.02	10 (62.5)	27 (45.8)	2.1 (0.6, 8.1)
Nickel ≥ 13.4	12 (75.0)	25 (42.4)	**6.8 (2.0, 22.8)**
Lead ≥ 1.2	9 (56.3)	28 (47.5)	1.5 (0.6, 3.7)
Antimony ≥ 0.14	10 (62.5)	27 (45.8)	2.0 (0.6, 6.3)
Selenium ≥ 0.87	6 (37.5)	32 (54.2)	0.5 (0.2, 1.4)
Tin ≥ 0.27	7 (43.7)	31 (52.5)	0.5 (0.2, 1.3)
Vanadium ≥ 0.02	9 (56.3)	28 (47.5)	1.4 (0.5, 3.4)
Zinc ≥ 96.8	9 (56.3)	28 (47.5)	1.6 (0.4, 7.0)

^a^ Adjusted for sex, age (continuous, years), body mass index (continuous, kg/m^2^), community area (adjacent, comparison), and clustering by school. *p* value < 0.1 shown in bold text. ACT = Asthma Control Test.

## Data Availability

The datasets used or analyzed during the current study are available from the corresponding author on reasonable request.

## Supplementary Materials

The following are available online at https://www.mdpi.com/article/10.3390/ijerph18147279/s1, Table S1: Spearman correlation coefficients for a suite of 17 trace and toxic metals measured in 75 toenail samples, Table S2: Associations between toenail metals concentrations and pulmonary function parameters among children with asthma (Hispanic only; *n* = 35), Table S3: Associations between toenail metals concentrations and percent predicted pulmonary function parameters among children with asthma (*n* = 39), Table S4: Associations be-tween toenail metals concentrations and percent predicted pulmonary function parameters among children with asthma (Hispanic only; *n* = 35), Table S5: Associations between toenail metal concentrations and asthma control among children with asthma (Hispanic only; *n* = 70).
