# Supplementary material for: Assessment of Metal Concentrations and Associations with Pulmonary Function among Children with Asthma in Chicago, Illinois
Source: Int J Environ Res Public Health. 2021 Jul 7;18(14):7279. doi: 10.3390/ijerph18147279 (PMC8307469; doi:10.3390/ijerph18147279)
Supplement: Supplementary file 1 [file ijerph-18-07279-s001.zip › ijerph-1167570-supplementary.pdf]

Supplemental Table S1. Spearman correlation coefficients for suite of 17 trace and toxic metals measured in 75 toenail samples

	Al	As	Cd	Co	Cr	Cu	Fe	Hg	Mn	Mo	Ni	Pb	Sb	Se	Sn	V	Zn
Al	1	0.23	0.24	0.28	0.28	0.41	0.24	0.29	0.29	0.28	0.06	0.34	0.34	0.09	0.15	0.37	0.14
As		1	0.48	0.59	0.51	0.48	0.42	0.12	0.56	0.47	0.36	0.47	0.64	-0.05	0.27	0.38	0.31
Cd			1	0.47	0.33	0.31	0.20	0.05	0.52	0.31	0.11	0.58	0.29	-0.03	0.30	0.53	0.16
Co				1	0.64	0.35	0.70	0.34	0.55	0.47	0.63	0.39	0.52	-0.11	0.15	0.41	0.15
Cr					1	0.41	0.51	0.31	0.44	0.57	0.49	0.38	0.36	-0.06	0.34	0.40	0.25
Cu						1	0.28	0.27	0.37	0.37	0.20	0.52	0.51	0.13	0.20	0.28	0.48
Fe							1	0.13	0.53	0.19	0.81	0.12	0.38	-0.19	-0.07	0.28	-0.01
Hg								1	0.07	0.31	0.21	0.16	0.17	0.23	0.29	0.06	0.25
Mn									1	0.33	0.38	0.33	0.48	-0.01	0.22	0.53	0.12
Mo										1	0.20	0.34	0.37	0.21	0.29	0.53	0.11
Ni											1	0.09	0.29	-0.11	-0.11	0.10	0.01
Pb												1	0.42	-0.07	0.25	0.44	0.31
Sb													1	-0.10	0.02	0.31	0.27
Se														1	0.35	0.07	0.38
Sn															1	0.16	0.35
V																1	0.22
Zn																	1

Supplemental Table S2. Associations between toenail metals concentrations and pulmonary function parameters among children with asthma (Hispanic only; n=35)

Exposure (µg/g)	ln (FEV1, L)[a]	ln (FVC, L)[a]	ln (FEV1:FVC)[a]	ln (FEF 25-75, L/sec)[a]
	β (SE)	β (SE)	β (SE)	β (SE)
Adjacent Community	-0.003 (0.07)	0.0005 (0.06)	-0.01 (0.05)	-0.05 (0.16)
P value	0.96	0.99	0.89	0.73
Aluminum ≥ 13.4	-0.04 (0.05)	0.01 (0.04)	-0.05 (0.03)	-0.13 (0.11)
P value	0.45	0.74	0.13	0.23
Arsenic ≥ 0.15	-0.004 (0.05)	0.03 (0.04)	-0.04 (0.03)	-0.13 (0.11)
P value	0.93	0.40	0.26	0.21
Cadmium ≥ 0.03	**-0.09 (0.04)**	-0.04 (0.04)	-0.04 (0.03)	-0.15 (0.10)
P value	**0.05**	0.27	0.17	0.15
Cobalt ≥ 0.05	-0.02 (0.05)	0.04 (0.04)	**-0.06 (0.03)**	-0.15 (0.11)
P value	0.62	0.34	**0.05**	0.16
Chromium ≥ 0.31	-0.03 (0.04)	-0.004 (0.04)	-0.03 (0.03)	-0.07 (0.10)
P value	0.44	0.92	0.32	0.48
Copper ≥ 4.5	**-0.12 (0.04)**	-0.05 (0.04)	**-0.07 (0.03)**	**-0.28 (0.10)**
P value	**0.004**	0.19	**0.02**	**0.004**
Iron ≥ 53.9	-0.002 (0.05)	-0.002 (0.04)	-0.0001 (0.03)	-0.04 (0.12)
P value	0.97	0.96	0.99	0.73
Mercury ≥ 0.03	-0.05 (0.05)	-0.03 (0.04)	-0.02 (0.04)	-0.08 (0.12)
P value	0.37	0.52	0.59	0.49
Manganese ≥ 1.1	**-0.14 (0.05)**	-0.06 (0.04)	**-0.08 (0.03)**	**-0.35 (0.10)**
P value	**0.002**	0.15	**0.02**	**0.001**
Molybdenum ≥ 0.02	-0.03 (0.04)	-0.06 (0.04)	0.03 (0.03)	0.03 (0.10)
P value	0.57	0.10	0.30	0.76
Nickel ≥ 15.2	0.04 (0.05)	**0.07 (0.04)**	-0.03 (0.03)	-0.05 (0.11)
P value	0.45	**0.07**	0.30	0.64
Lead ≥ 1.2	-0.02 (0.05)	-0.07 (0.04)	0.05 (0.04)	0.10 (0.12)
P value	0.73	0.12	0.18	0.42
Antimony ≥ 0.14	-0.01 (0.05)	-0.001 (0.04)	-0.01 (0.04)	-0.08 (0.12)
P value	0.84	0.98	0.75	0.51
Selenium ≥ 0.88	-0.07 (0.05)	**-0.12 (0.04)**	0.05 (0.04)	0.04 (0.12)
P value	0.18	**0.003**	0.16	0.72
Tin ≥ 0.27	0.04 (0.05)	0.01 (0.04)	0.04 (0.03)	0.14 (0.11)
P value	0.36	0.88	0.25	0.19
Vanadium ≥ 0.02	**-0.09 (0.04)**	**-0.08 (0.04)**	-0.004 (0.03)	-0.10 (0.10)
P value	**0.04**	**0.02**	0.89	0.35
Zinc ≥ 98.8	-0.05 (0.05)	-0.04 (0.04)	-0.01 (0.03)	-0.06 (0.11)
P value	0.33	0.34	0.84	0.57

[a] Adjusted for sex, age (continuous, years), body mass index (continuous, kg/m^2), height (continuous, centimeters), community area (adjacent, comparison), and school.
P value <0.1 shown in bold text.

Supplemental Table S3. Associations between toenail metals concentrations and percent predicted pulmonary function parameters among children with asthma (n=39)

Exposure (μg/g)	FEV1 %[a]	FVC %[a]	FEV1:FVC %[a]	FEF 25-75%[a]
	β (SE)	β (SE)	β (SE)	β (SE)
Adjacent Community	0.58 (5.4)	-2.8 (4.7)	2.8 (4.0)	2.1 (12.3)
P value	0.91	0.55	0.47	0.86
Aluminum ≥ 13.4	-3.5 (4.0)	0.53 (3.5)	-3.8 (2.9)	-6.9 (9.2)
P value	0.39	0.88	0.19	0.45
Arsenic ≥ 0.15	-1.5 (3.9)	2.0 (3.4)	-3.1 (2.8)	-11.5 (8.8)
P value	0.70	0.57	0.27	0.19
Cadmium ≥ 0.03	-6.3 (3.9)	-3.5 (3.4)	-2.4 (2.9)	-11.4 (8.9)
P value	0.11	0.30	0.40	0.20
Cobalt ≥ 0.05	-0.81 (4.1)	3.7 (3.5)	-4.6 (2.9)	-5.0 (9.2)
P value	0.84	0.29	0.11	0.59
Chromium ≥ 0.31	-3.2 (3.9)	0.48 (3.4)	-3.2 (2.8)	-6.1 (8.8)
P value	0.41	0.89	0.25	0.49
Copper ≥ 4.5	**-9.0 (3.8)**	-3.7 (3.4)	**-5.4 (2.8)**	**-20.6 (8.5)**
P value	**0.02**	0.29	**0.05**	**0.01**
Iron ≥ 53.9	-1.3 (3.9)	0.12 (3.3)	-1.4 (2.8)	-7.2 (8.7)
P value	0.74	0.97	0.63	0.41
Mercury ≥ 0.03	-0.61 (4.0)	-1.2 (3.5)	0.77 (3.0)	2.1 (9.2)
P value	0.88	0.72	0.79	0.82
Manganese ≥ 1.1	**-9.1 (3.9)**	-3.3 (3.6)	**-5.4 (2.9)**	**-24.9 (8.6)**
P value	**0.02**	0.36	**0.06**	**0.004**
Molybdenum ≥ 0.02	-3.0 (4.0)	**-6.2 (3.3)**	3.3 (2.8)	0.85 (9.0)
P value	0.44	**0.06**	0.24	0.92
Nickel ≥ 15.2	2.0 (4.0)	**6.4 (3.3)**	-4.5 (2.8)	-7.3 (9.0)
P value	0.62	**0.05**	0.11	0.41
Lead ≥ 1.2	-0.28 (4.2)	-5.2 (3.5)	**5.3 (2.9)**	7.6 (9.4)
P value	0.94	0.14	**0.07**	0.42
Antimony ≥ 0.14	-0.41 (4.4)	2.3 (3.8)	-1.5 (3.2)	-6.8 (10.0)
P value	0.92	0.55	0.63	0.49
Selenium ≥ 0.88	-3.6 (4.3)	**-8.8 (3.5)**	**5.3 (3.0)**	4.8 (9.8)
P value	0.40	**0.01**	**0.08**	0.62
Tin ≥ 0.27	1.7 (3.8)	-2.3 (3.3)	4.2 (2.7)	8.3 (8.6)
P value	0.66	0.48	0.12	0.33
Vanadium ≥ 0.02	**-8.6 (3.8)**	**-8.0 (3.2)**	-0.39 (2.9)	-8.2 (9.0)
P value	**0.02**	**0.01**	0.89	0.36
Zinc ≥ 98.8	-4.4 (4.2)	-2.9 (3.6)	-1.3 (3.1)	-8.5 (9.5)
P value	0.29	0.42	0.67	0.37

[a] Adjusted for body mass index (continuous, kg/m^2), height (continuous, centimeters), community area (adjacent, comparison), and school.
P value <-0.1 shown in bold text.

Supplemental Table S4. Associations between toenail metals concentrations and percent predicted pulmonary function parameters among children with asthma (Hispanic only; n=35)

Exposure (µg/g)	FEV1 %[a]	FVC %[a]	FEV1:FVC %[a]	FEF 25-75%[a]
	β (SE)	β (SE)	β (SE)	β (SE)
Adjacent Community	2.1 (5.9)	-0.65 (5.0)	2.5 (4.4)	1.7 (13.6)
P value	0.72	0.90	0.56	0.90
Aluminum ≥ 13.4	-3.1 (4.3)	1.5 (3.7)	-4.2 (3.2)	-6.8 (10.0)
P value	0.47	0.69	0.18	0.50
Arsenic ≥ 0.15	0.07 (4.3)	3.6 (3.7)	-2.9 (3.2)	-10.3 (10.0)
P value	0.99	0.32	0.36	0.30
Cadmium ≥ 0.03	-6.6 (4.1)	-3.6 (3.6)	-2.6 (3.2)	-11.3 (9.8)
P value	0.11	0.31	0.42	0.24
Cobalt ≥ 0.05	-0.37 (4.3)	4.6 (3.6)	-4.9 (3.1)	-4.5 (10.0)
P value	0.93	0.20	0.11	0.65
Chromium ≥ 0.31	-3.3 (4.2)	-0.002 (3.6)	-2.9 (3.1)	-5.6 (9.7)
P value	0.43	0.99	0.35	0.56
Copper ≥ 4.5	**-9.9 (4.0)**	-4.9 (3.6)	**-5.3 (3.1)**	**-21.6 (9.4)**
P value	**0.01**	0.17	**0.09**	**0.02**
Iron ≥ 53.9	-1.4 (4.2)	-0.74 (3.6)	-0.78 (3.1)	-6.7 (9.7)
P value	0.74	0.84	0.80	0.49
Mercury ≥ 0.03	-0.85 (4.5)	-0.54 (3.8)	0.03 (3.3)	0.80 (10.4)
P value	0.85	0.89	0.99	0.94
Manganese ≥ 1.1	**-11.5 (4.3)**	-5.7 (3.9)	**-5.7 (3.4)**	**-28.9 (9.9)**
P value	**0.008**	0.15	**0.09**	**0.004**
Molybdenum ≥ 0.02	-2.6 (4.2)	-5.4 (3.5)	3.3 (3.1)	0.44 (9.8)
P value	0.54	0.12	0.29	0.96
Nickel ≥ 15.2	2.5 (4.3)	**6.6 (3.5)**	-4.2 (3.1)	-6.8 (9.9)
P value	0.56	**0.06**	0.17	0.49
Lead ≥ 1.2	0.35 (4.6)	-5.3 (3.8)	**6.2 (3.3)**	10.2 (10.6)
P value	0.94	0.17	**0.06**	0.34
Antimony ≥ 0.14	-2.1 (4.7)	0.77 (4.0)	-1.9 (3.5)	-9.5 (10.9)
P value	0.66	0.85	0.58	0.38
Selenium ≥ 0.88	-5.1 (4.5)	**-10.8 (3.5)**	5.5 (3.3)	3.0 (10.8)
P value	0.26	**0.002**	0.10	0.78
Tin ≥ 0.27	4.4 (4.2)	-0.21 (3.6)	**5.1 (3.0)**	10.9 (9.7)
P value	0.29	0.95	**0.09**	0.26
Vanadium ≥ 0.02	**-8.7 (4.0)**	**-8.0 (3.4)**	-0.36 (3.2)	-7.9 (9.8)
P value	**0.03**	**0.02**	0.91	0.42
Zinc ≥ 98.8	-4.1 (4.5)	-3.1 (3.9)	-0.77 (3.4)	-8.2 (10.6)
P value	0.37	0.42	0.82	0.43

[a] Adjusted for body mass index (continuous, kg/m^2), height (continuous, centimeters), community area (adjacent, comparison), and school.
P value <-0.1 shown in bold text.

Supplemental Table S5. Associations between toenail metal concentrations and asthma control among children with asthma (Hispanic only; n=70)

**Exposure (μg/g)**	**Uncontrolled ACT ≤ 19 (n=16)**	**Controlled ACT >19 (n=55)**	**OR (95% CI)[a]**
	**N (%)**	**N (%)**	
Adjacent Community	9 (56.3)	26 (48.1)	1.3 (0.4, 4.5)
Aluminum ≥ 12.3	10 (62.5)	23 (42.6)	2.3 (0.9, 6.0)
Arsenic ≥ 0.15	10 (62.5)	24 (44.4)	1.8 (0.6, 5.9)
Cadmium ≥ 0.03	9 (56.3)	24 (44.4)	1.5 (0.5, 4.7)
Cobalt ≥ 0.04	10 (62.5)	23 (42.6)	2.5 (0.7, 9.6)
Chromium ≥ 0.29	9 (56.3)	26 (48.1)	1.3 (0.2, 10.0)
Copper ≥ 4.5	12 (75.0)	24 (44.4)	**4.5 (1.0, 20.4)**
Iron ≥ 51.2	11 (68.8)	25 (46.3)	3.2 (0.8, 12.2)
Mercury ≥ 0.03	9 (56.3)	25 (46.3)	1.8 (0.4, 6.9)
Manganese ≥ 1.1	9 (56.3)	26 (48.1)	1.1 (0.3, 4.3)
Molybdenum ≥ 0.02	10 (62.5)	22 (40.7)	2.6 (0.7, 8.9)
Nickel ≥ 13.4	12 (75.0)	23 (42.6)	**7.3 (1.9, 28.2)**
Lead ≥ 1.2	9 (56.3)	25 (46.3)	1.6 (0.6, 4.3)
Antimony ≥ 0.14	10 (62.5)	23 (42.6)	2.1 (0.7, 6.6)
Selenium ≥ 0.87	6 (37.5)	28 (51.8)	0.5 (0.1, 1.6)
Tin ≥ 0.27	7 (43.7)	27 (50.0)	0.6 (0.2, 1.3)
Vanadium ≥ 0.02	9 (56.3)	24 (44.4)	1.5 (0.7, 3.3)
Zinc ≥ 96.8	9 (56.3)	26 (48.1)	1.6 (0.4, 6.9)

[a] Adjusted for sex, age (continuous, years), body mass index (continuous, kg/m^2), community area (adjacent, comparison), and clustering by school.
P value <0.1 shown in bold text.
